# The Relation of Tests of Scientific Reasoning to Each Other and to Tests of General Intelligence

**DOI:** 10.3390/jintelligence7030020

**Published:** 2019-08-30

**Authors:** Robert J. Sternberg, Chak Haang Wong, Karin Sternberg

**Affiliations:** Department of Human Development, Cornell University, Ithaca, NY 14853, USA

**Keywords:** drawing conclusions, general intelligence, generating hypotheses, generating experiments, intelligence, scientific reasoning

## Abstract

We conducted two studies to replicate and extend, as well as test, the limits of previous findings regarding an apparent disconnect between scientific-reasoning skills in psychological science, on the one hand, and scores on standardized tests of general intelligence, on the other. In Study 1, we examined whether this disconnect would extend beyond psychological science to additional sciences as well, such as nutrition and agriculture. The results did indeed extend, suggesting that scientific reasoning across various natural sciences is comparable to scientific reasoning in psychological science, but different in kind from the reasoning required on conventional standardized tests. In Study 2, we examined whether these findings were linked to the format of presentation of scientific problems. Whereas real scientific-reasoning problems are open-ended, standardized tests tend to use multiple-choice format. We discovered that using multiple-choice format did indeed result in an apparently closer relation of the scientific-reasoning tests to two of the conventional ability measures (SAT Reading and Number Series) but not to two other tests (Letter Sets and SAT Math). Thus, one can increase the correlations of scientific-reasoning tests with at least some standardized ability tests but at the cost of content validity and ecological validity.

## 1. Introduction

Graduate schools use a variety of criteria to determine whom to admit. But graduate admissions in scientific fields, and in many other fields as well, rely, often quite substantially, on scores of candidates on the Graduate Record Examination (GRE) [1]. Some researchers, however, have argued that tests such as the GRE (as well as the SAT, the ACT, and other related tests) assess a relatively narrow band of analytical-thinking skills [2,3,4], such as inferential reasoning, similar to those on IQ tests. Tests like the ACT, SAT, and GRE provide reasonably good measures of general cognitive ability [5,6,7], but they do not measure the research and teaching skills that are particularly relevant to success in science [8,9].

In previous work, investigators have sought to expand the range of assessment for graduate admissions in psychological science and related disciplines, see References [8,9]. Our research was motivated in part by a study R. J. Sternberg did with Wendy Williams more than 20 years ago [10], which showed that, for the select population of graduate students in psychology at Yale University, the GRE was not a particularly successful predictor of performance, measured in terms of first-year course grades, in a graduate psychology program (at Yale). In particular, Sternberg and Williams [10] looked into the validity of the GRE for predicting various aspects of graduate performance in the Psychology Department at Yale University. As criteria, they used (a) grades in first-and second-year courses, (b) ratings by professors of student dissertations, and (c) ratings by professors of students’ abilities of four kinds: analytical, creative, practical, and teaching (the last of which also involves the other three). The GRE was discovered to be validly predictive only of grades in the first year of graduate school, with the notable exception of the analytical section. It was also shown to be validly if weakly predictive of some additional criteria, such as ratings by professors of various aspects of student performance, but for men only. In particular, Sternberg and Williams found the predictive validity of the GRE, in their sample, to be 0.18 for the verbal test, 0.14 for the mathematical test, 0.17 for the analytical test, and 0.37 for the psychology subject-matter test, in predicting first-year grades. Sternberg and Williams did not, however, propose any specific kind of alternative or supplementary test.

A more positive view regarding the predictive validity of the GRE can be found elsewhere ([11]; see also Reference [8] for a related brief review of this literature). The investigators performed a meta-analysis assessing the validity of the GRE for predicting graduate grade-point average across a variety of graduate fields. Their meta-analysis showed correlations of 0.34 for the GRE verbal, 0.38 for the GRE mathematical, 0.36 for the GRE analytical, and 0.45 for the GRE subject-matter tests. However, these correlations were corrected for both restriction of range and attenuation, so that they were not really comparable to those obtained by Sternberg and Williams. Correlations from this study of the GRE with other relevant graduate-level criteria were not as strong. For example, the obtained validity coefficient vis a vis research productivity was 0.09 for the GRE Verbal, 0.11 for the GRE Mathematical, and 0.21 for the GRE subject-matter test (corrected for restriction of range). Correlations of the GRE with the amount of time it took to complete the degree were 0.28 for the verbal test, −0.12 for the mathematical test, and −0.02 for the subject-matter test. Other research [12,13] found further evidence that standardized tests can validly predict graduate students’ success.

Other investigators also have examined the predictive validity of the GRE. For example, one investigation [14] examined the predictive validity of the GRE in predicting first-year grades in psychology. The investigator found validity coefficients of 0.18 for the verbal test, 0.19 for the mathematical test, and 0.32 for the analytical test. Further work [15] examined the predictive validity of the GRE in predicting graduate psychology grades in the first year of study. The validity coefficients resulting from this study were 0.26 for the verbal test, 0.25 for the mathematical test, 0.24 for the analytical test, and 0.36 for the subject-matter test.

Further studies on the “new GRE” published by the Educational Testing Service can be found elsewhere [16]. The new GRE is much like the old GRE. It contains verbal and mathematical reasoning sections, plus a writing section.

A further consideration is that data compiled by the Educational Testing Service, the publisher of the GRE, suggests notable gaps between the performances of males versus females, on the verbal test and especially the mathematical test, and in both cases favoring the males [17]. Yet females tend to perform better in courses than males. A referee of this article has suggested that GRE underprediction for females in course performance may be, in part, because females are more conscientious, on average, than males, and conscientiousness predicts grade-point-average GPA [18]. This suggestion would need further empirical tests to ascertain its validity.

These differences appear to be sustained across a range of racial/ethnic groups. However, there is no evidence that males perform better than females in their graduate-school work. The gap in GRE test results, therefore, must be a matter of at least some concern. In addition, there also are racial and ethnic group differences in GRE test scores [19]. These results possibly might suggest some kinds of adverse impacts for the test, at least to the extent that one might wish all groups to enter the testing situation with equal opportunities to score well.

Our own studies following up on Sternberg and Williams were based on the simple notion that the best predictor of future behavior of any kind is past behavior of the same kind. Hence, we asked what particular skills future psychological scientists will actually need in graduate school and later when (or if) they take scientifically-oriented jobs. The two main things psychological scientists do are research and teaching. Thus, our new measures were designed to assess reasoning about research and teaching in the context of psychological science.

The measures required, in a psychological-scientific context, students to (a) generate alternative hypotheses, (b) generate experiments, (c) draw conclusions, (d) review articles, and (e) evaluate teaching, among other things. For example, students might be presented with an experiment, be given a hypothesis about why the results were obtained, and then be asked to generate alternative hypotheses regarding why that result came about. Or they might be presented with a video of a professor teaching a psychology lesson, and then be asked to specify what the teacher is doing sub-optimally (from a pedagogical point of view) in teaching the lesson. 

Here are three quoted examples of the actual problems we used regarding reasoning about research.

## 2. Generating Hypotheses

Marie is interested in child development. One day, she notices that whenever Laura’s nanny comes in to pick up Laura from nursery school, Laura starts to cry. Marie reflects upon how sad it is that Laura has a poor relationship with her nanny.

What are some alternative hypotheses regarding why Laura starts to cry when she is picked up from nursery school by the nanny?

## 3. Generating Experiments

Ella, a senior in college, observes that her roommate tends to perform better on an exam if she has had a cup of coffee beforehand. Ella hypothesizes that drinking coffee before taking an exam will significantly increase one’s exam performance. However, Ella does not know how to test this hypothesis.

Please suggest an experimental design to test this hypothesis and describe the experiment in some detail. Assume you have the resources you need to be able to do the experiment (e.g., access to students and their academic records, sufficient funds to pay subjects, etc.).

## 4. Drawing Conclusions

Bill was interested in how well a new program for improving mathematical performance worked. He gave 200 students a pretest on their mathematical knowledge and skills. He then administered the new program to them. After administering the program, he gave the same 200 students a posttest that was equal in difficulty and in all relevant ways comparable to the pretest. He found that students improved significantly in performance from pretest to posttest. He concluded that the program for improving mathematical performance was effective.

Is this conclusion correct? Why or why not?

These measures were selected on the basis of the purported processes of scientific reasoning, not only in psychology but in other scientific disciplines, as presented in the large majority of textbooks in introductory psychology, as well as in textbooks on experimental-methods. For instance, one researcher [20] characterized three of these processes of scientific reasoning as “come up with alternative hypotheses”, “design an experiment”, and “see which outcome you get, and therefore which hypotheses survived” (p. 41). Different textbooks have different wording but in almost all of the texts, including one we have used [21], three of the mental processes correspond to what we refer to in our studies as “generating hypotheses”, “generating experiments”, and “drawing conclusions”. Viewed in terms of the theory of successful intelligence, the processes of generating hypotheses and generating experiments are largely in the creative domain, whereas drawing conclusions is largely in the analytical domain. All are further relevant to the practical domain, that is, the practice of doing science.

Rated performance on these measures was highly reliable and was compared with performance on tests of fluid intelligence (Number Series and Letter Sets) and also on standardized tests (SAT/ACT) and undergraduate GPAs.

The main findings were that (a) the various measures of Scientific Reasoning and Reasoning about Teaching clustered together correlationally and hence factorially; (b) the inductive-reasoning tests and the standardized tests also clustered together (as they are all, at some level, measures of general intelligence); and (c) our tests did NOT cluster with the inductive-reasoning or standardized tests, suggesting that they measure a different construct. Moreover, (d) our tests did not show the gender gap that is commonly found on many standardized tests. Details can be found in the original articles, see [8,9].

We thought it was interesting that our measures of reasoning about research and about teaching clustered together factorially, suggesting that many of the same analytical, creative, and practical skills that investigators apply to research can be applied to teaching—if the researchers expend the effort to think about their teaching in the same way they do about their research.

We suggested that these results are important because, first, admission to graduate school in psychological science is more competitive than ever; second, we need excellent scientists more than ever; and yet, third, we may be admitting and rejecting, in some cases, the wrong people. The measures we typically use to assess young people focus almost entirely on memory and abstract-analytical skills [22,23], to the exclusion of the creative and practical skills students eventually will also need to succeed in their work. We believe our results are consistent with this view—that if we want to admit students who later will become excellent researchers and teachers, we should gather an early assessment of their skills for success in their future discipline in research and teaching. 

The goal in our research is not to “dump” the GRE and related tests. We recognize that students who lack sufficient levels of the kind of academic preparation measured by these tests (the Scientific Reasoning tests) may have trouble simply meeting the academic requirements of many graduate programs. They also may be challenged in their later research and teaching. But we believe conventionally measured cognitive and academic skills, while necessary, at some level, for graduate success, are by no means sufficient. And moreover, they are necessary at more modest levels than many of the competitive programs require. That is, to succeed in a competitive program, what really matters most ultimately is not whether one has top-tier scores on a standardized test, but rather, whether one will be able to succeed in research and ultimately in teaching. Some students applying to graduate school have had research experience, but the quantity and quality of that experience often depend on the availability of opportunities in the undergraduate institution one attended. Our assessments, in contrast, are relevant and could be potentially accessible to students in any institution.

Our tests were based on Sternberg’s [23,24] theory of successful intelligence. According to the theory, intelligence can be understood, in part, in terms of information-processing components that, in combination, constitute intelligent thought and give rise to intelligent behavior.

Metacomponents are higher order executive processes that are utilized to plan what one is going to do, to monitor it while it is being done, and then to evaluate it after it is completed. In a scientific context, metacomponents might be used to formulate a strategy for solving a particular problem, for example, how to design a set of experiments to test a particular scientific hypothesis or set of hypotheses. Performance components are utilized in order to implement the strategies the metacomponents constructed. An example of a performance component would be inferring relations, for example, inferring how the outcomes of a given test of statistical significance best should be interpreted. Knowledge-acquisition components are utilized to learn how to solve particular kinds of problems in the first place. An example of a knowledge-acquisition component would be selective comparison, whereby an individual tries to figure out what particular information stored in long-term memory is relevant to, and useful for, solving a problem. An example would be retrieving the knowledge needed to perform an appropriate and useful test of statistical significance in a given experiment.

Our current program of research follows up previous research [8,9], as well as on other research we have done on admissions at other levels and for other kinds of specializations see, e.g., References [4,22]. In these studies, metacomponents are used to figure out exactly what problem is being presented, performance components to solve the problems, and knowledge-acquisition components to understand the material as presented in the problem scenarios.

Our current studies extend our previous work we have done in two ways. These two ways are designed to determine both how replicable and how generalizable our results are, both of which are important considerations in science.

First, we sought to extend the results from previous studies beyond the domain of psychological science to include a broader range of sciences. On the one hand, there is no reason to expect the basic processes of scientific reasoning to be different from one science to another. On the other hand, many scientists do not consider psychology to be a “real science” [25]. Rather, they view psychology as some kind of “soft science” whose methods and the reasoning that underlie them are, at best, quasi-scientific. Hence, it is a legitimate question whether the results we obtained for psychological-science items would extend beyond psychological science. We expected to get the same results, regardless of the science at issue, as psychological-scientific reasoning involves the same kinds of thinking as in other natural sciences, such as formulating alternative hypotheses, generating experiments to test hypotheses, and drawing conclusions from empirical data.

Second, we sought to address a concern we had—whether the low correlations we obtained between our measures and more conventional standardized tests might be, in part, a function of the mode of testing—in particular, the multiple-choice format that characterizes most standardized tests versus the extended free-response format that characterizes our tests. Some of our past results suggest that, when tests are given in multiple-choice format, they tend to load more heavily on the analytical factor measured by *g-*based tests [26,27,28]. Our second study specifically addressed the question of what would happen if the scientific-reasoning tests were presented in multiple-choice format (a format that, in the real world of science, never presents itself). We expected that using multiple-choice format would bring our tests closer, correlationally and factorially, to conventional tests of *g-*based analytical skills. However, we also believe that, in using such a format, one loses part of what one originally seeks to measure, namely, how scientists will function when presented with problems for which there are never explicitly presented multiple choices available. Thus, one gains a closer relation with standardized tests at the expense of content validity and ecological validity.

## 5. Study 1

Study 1 extended our previous results [8,9] by examining whether the kinds of results obtained for scientific-reasoning problems in psychological science also would be obtained for scientific-reasoning problems in other domains of science. In other words, do the results replicate and do they generalize? It has become more and more clear that many findings in psychological science do not replicate or generalize well [29], so it is especially important both to assess replicability and also to examine the limits of generalization of our findings.

### 5.1. Method

#### Participants

A total of 88 university students (2 graduate students and 86 undergraduate students) participated in the data collection. In all, 57 of them were female, and 31 of them were male. Students were enrolled in 36 different majors, with 5 students not indicating their majors, and 5 students reporting double majors. The participants’ average age was 20.11 years, with a standard deviation of 1.23. Moreover, 43 (48.9%) out of the 88 students indicated that they are contemplating a career in STEM (science-technology-engineering-mathematics)-related field, and 33 (37.5%) out of the 88 students indicated that they had taken research-methods courses prior to this study.

### 5.2. Materials

There were three categories of tasks: Psychometric assessments, which include a Letter-Sets test and a Number-Series test; Scientific-Reasoning tasks, which include three sections (Generating Hypotheses, Designing Experiments, and Drawing Conclusions); and a demographic questionnaire. The assessments were informally piloted on a small sample of students before being used in our research. The psychometric assessments were timed for 7 min each and the scientific-reasoning items took roughly 30–45 min in total.

The psychometric assessments were adopted from our previous work as mentioned above, see Reference [8], and were mainly measuring fluid intelligence. They were recorded and measured based on the number of correct answers; each correct answer was granted one point.

For each section of the Scientific-Reasoning tasks, we created three new items in connection with general science and related disciplines, and we retrieved two old items (in connection with psychology and related disciplines) from our previous work as mentioned above, see Note [8]. To remind readers, old items were limited to psychological science and new items were more broadly ranging in the sciences. There was a total of five items for each section. In terms of scoring, for Generating Hypotheses, one point was granted for a valid and reasonable hypothesis. For Designing Experiments and Drawing Conclusions, each item was judged on 5-point scales (1 = poorly answered and 5 = thoroughly answered).

#### 5.2.1. Letter Sets

The Letter Sets test had 15 problems as used previously in our previous work [8,9]. Each problem contained five sets of four letters. The task was to cross out one set of letters that was different than the rest of the sets. An example would be among KLOP, HOMT, PLIS, MORW, and OLSP, PLIS should be crossed out because it is the only set of letters that do not contain the letter “O”. If a participant crosses out the correct answer, PLIS, then he/she would be granted one point. In previous research, we corrected for guessing but the corrected scores were almost perfectly correlated with the uncorrected scores so we did not correct here.

#### 5.2.2. Number Series

The number-series tests had 18 problems, and each problem presented a series of numbers with a blank in the end indicating the next number. Items were used earlier in our previous work [8,9]. The participants needed to find the pattern of the numbers in order to fill in what the next number would be in the series. For example, in the series of 8, 8, 16, 16, 24, the next number would be 24 because each number is repeated once. One point was granted for each correct answer. As for Letter-Sets, in previous research, we corrected for guessing but the corrected scores were almost perfectly correlated with the uncorrected scores so we did not correct here.

#### 5.2.3. Generating Hypotheses

For this test and the other Scientific-Reasoning tests described below, all items are quoted as they were used in the studies described in this article. In other words, they were the items participants actually saw.

A short scenario was described, and the participants were asked to generate as many alternative hypotheses as they could at the moment. For example:

“Jasper is interested in the function of water when growing plants. He adds 50 mL of water to the earth in which half of his plants are growing and 150 mL of water to the earth in which the other half of his plants are growing. He notices that plants with the 150 mL of water grow taller than those with the 50 mL of water and claims that water helps the plants to grow even more.

What are some alternative hypotheses regarding why the plants with 150 mL of water grow taller?”

#### 5.2.4. Designing Experiments

A short description of a hypothesis was presented, and the participants were asked to design an experiment that could adequately test the hypothesis. For example:

“April is interested in making her seeds sprout faster. She hypothesizes that higher temperatures can make the seeds sprout faster and earlier. However, April is not sure how to properly design an experiment to test this hypothesis.

Please suggest an experimental design to test this hypothesis and describe the experiment in some detail. Assume you have the resources you need to be able to do the experiment.”

#### 5.2.5. Drawing Conclusions

A short description of an experiment’s results was presented, and the participants were asked to explain whether or not the conclusions drawn from the results were valid. For example:

“Tony wanted to see whether exercising for 30 min per day would reduce cholesterol levels. He had 10 subjects exercise for at least 30 min per day for three weeks and 10 subjects not exercise at all. He also controlled the number of calories all subjects consumed daily. He found that cholesterol levels were reduced for those who did the exercise. He concluded that exercising reduces cholesterol levels and thus lowers the risk of having heart disease.

Is the conclusion correct? Why or why not?”

#### 5.2.6. Demographic Questionnaire

A demographic questionnaire assessed variables including gender, age, major, GPAs, self-reported SAT and ACT scores, number of lab course taken, number of math and science courses taken, whether or not they have taken research-methods courses, and whether or not they were contemplating career in a science-related field. Past research has indicated that self-reported SAT and ACT scores tend to have high validity [30].

### 5.3. Design

In the analysis of variance, the dependent variables were standardized test scores, and the independent variable was participant sex. In the correlational analyses, the dependent variables were standardized test scores, and the independent variables were our scientific-reasoning test scores.

### 5.4. Procedure

The participants were first asked to read and sign the informed consent form. Then, a set of assessments in the form of questionnaires were handed to the participants. The assessments were arranged in the following order: Letter Sets, Number Series, Generating Hypotheses, Generating Experiments, Drawing Conclusions, and a brief demographic questionnaire. The first two assessments had a seven-minute time limit, and they were timed by an experimenter with a stopwatch. After completion of the Number Series test, the participants were asked to read the instructions carefully and finish the rest of the questionnaires at their own pace. After completion of the demographic questionnaire, a debriefing form was presented to the participants when they handed the package back to the experimenter. The entire session lasted about 1.5 h.

### 5.5. Results

All data analyses were done via SPSS. Table 1 shows means and standard deviations for the various measures used in the study. Cronbach alpha reliabilities of the scales were 0.61 for Letter Sets, 0.66 for Number Series, 0.88 for Generating Hypotheses, 0.71 for Generating Experiments, 0.48 for Drawing Conclusions, and 0.84 for the combined scientific-reasoning measures (Generating Hypotheses, Generating Experiments, Drawing Conclusions, combined).

#### 5.5.1. Gender Analysis

When results were analyzed by gender, none of the differences in means was statistically significant.

#### 5.5.2. Correlations

Table 2 shows a complete intercorrelation matrix for our variables. The main things to notice are that (a) two of the three correlations between our tests of Scientific Reasoning were statistically significant (Generating Hypotheses and Drawing Conclusions at 0.33, and Generating Experiments and Drawing Conclusions at 0.43) while one was not (Generating Hypotheses and Generating Experiments at 0.09); (b) the two psychometric tests, Letter Sets and Number Series, were significantly correlated at 0.37; (c) Letter Sets was significantly correlated with both SAT Reading and SAT Math (0.29 and 0.28 respectively); (d) Number Series was correlated significantly with SAT Math (0.44) but not with SAT Reading (0.18); (e) none of our Scientific-Reasoning measures showed any significant correlations with either Letter Sets or Number Series; and (f) our Scientific-Reasoning measures, combined, showed statistically significant *negative* correlations with both SAT Reading and SAT Math (−0.21 and −0.29, respectively).

#### 5.5.3. Factor Analyses

Table 3 shows the varimax-rotated principal-components loadings for the new scientific-reasoning tests as well as for the tests of fluid intelligence (*g-f*). The components reveal two clear factors, one for our tests of Scientific Reasoning and one for the tests of fluid intelligence. Table 4 shows that principal-factor analysis revealed comparable results.

Table 5 shows the varimax-rotated principal-components loadings for the new Scientific- Reasoning tests as well as for the university-level tests of academic skills. The pattern is much like that in Table 3. Our tests of Scientific Reasoning load on one factor, whereas the tests of academic skills load on another. Table 6 shows comparable results using principal-factor analysis.

Table 7 shows the varimax-rotated principal-components loadings for both the new and the old Scientific-Reasoning items plus the tests of fluid intelligence plus the tests of academic skills. Both our new items and our old items loaded on the same factor, suggesting they measure largely the same skills. The tests of fluid intelligence and of academic skills loaded on the second and third factors, with the third factor apparently being a quantitative factor (including Number Series and Math SAT). The rotated principal-factor loadings in Table 8 show a similar but less clear pattern.

### 5.6. Discussion

The results of this experiment were consistent with our hypotheses. Similar or identical processes of reasoning appear to be used for psychological-scientific reasoning and for natural-scientific reasoning in general. Indeed, the results for this work were fully comparable to those for the earlier work done by our previous studies (Sternberg & Sternberg, 2017; Sternberg, Sternberg, & Todhunter, 2017). The results suggest that scientific reasoning of the kinds we have assessed in this experiment, whether for psychological science or science in general, measure skills other than those measured by tests of fluid and crystallized abilities. As a result, graduate programs seeking strong scientific reasoners would need to supplement conventional tests with tests that specifically measure the skills that will be relevant not only in graduate training but also in a future scientific career.

## 6. Study 2

Study 2 examined the extent to which the results from Study 1 would generalize to the same problems presented in multiple-choice rather than free-response format. It was predicted that using such a format would increase correlations of our Scientific-Reasoning tests with some of the standardized tests of intellectual abilities because of the overlap in format. But such overlap actually would lower content and ecological validity of the items, because in real-world science, scientific problems are not presented in multiple-choice format.

### 6.1. Method

#### Participants

A total of 139 undergraduate students (44 freshmen, 48 sophomores, 31 juniors, and 16 seniors) participated in the data collection. In all, 98 of them were female and 41 of them were male. Students were enrolled in 43 different majors, with 6 students not indicating their majors and 8 students reporting double majors. The participants’ average age was 19.22 years, with a standard deviation of 0.97. Moreover, 90 (64.75%) indicated that they are contemplating a career in a STEM-related field, and 45 (32.37%) indicated that they had taken research-methods courses prior to this study.

### 6.2. Materials

There were three categories of tasks: psychometric assessments, which include the Letter Sets test and the Number-Series test; Scientific-Reasoning tasks, which include three sections: Generating Hypotheses, Designing Experiments, and Drawing Conclusions; and a demographic questionnaire. The assessments were informally piloted on a small sample of students before being used in our research. The psychometric assessments were timed for 7 min each and the scientific-reasoning items took roughly 30–45 min in total.

The psychometric assessments were adopted in our previous study and mainly measured fluid intelligence. They were scored based on the number of correct answers, and each correct answer was granted one point. The total score was 15 for the Letter-Sets test and 18 for the Number-Series test.

For each section of the Scientific-Reasoning test, there was a total of 6 items, in which 3 of them were multiple choice and 3 of them were free-response. Two forms of surveys, Form A and Form B, were randomly assigned across participants. The two forms were different in the way that, for the Scientific-Reasoning test section, items that were in multiple-choice format in Form A were shown in free-response format in Form B, and vice versa. In other words, the exact same item stem was shown with different response formats between Form A and Form B. Each participant saw a given stem in only one form, either as multiple-choice or as free-response. In terms of scoring, one point was granted if a multiple-choice item was answered correctly. For judging the free-responses, as was previously the case, one point was granted for a valid hypothesis in Generating Experiments, and a 5-point scale was used for Designing Experiments and Drawing Conclusions (1 = poorly answered through 5 = very well answered). Thus, for each section, the total score was 3 for the multiple-choice items.

#### 6.2.1. Letter Sets

The Letter-Sets items were the same as in Study 1.

#### 6.2.2. Number Series

The Number-Series items were the same as in Study 1.

#### 6.2.3. Generating Hypotheses

A short scenario was described, and for the multiple-choice items, participants were asked to choose the most plausible alternative hypothesis out of the five options.

For the free-response items, participants were asked to generate as many alternative hypotheses as they could at the moment. These items were as in Study 1.

Here, however, is an example of a Generating-Hypotheses item converted to multiple-choice format:

“Jasper is interested in the function of water when growing plants. The plants are randomly sorted into two different plots. He adds 50 mL of water to the earth in which half of his plants are growing and 150 mL of water to the earth in which the other half of his plants are growing. He notices that plants with the 150 mL of water grow taller than those with the 50 mL of water and claims that extra water helps the plants to grow even more.

Which of the following is the MOST plausible alternative hypothesis for why the plants with 150 mL of water grew taller?

- It may be that the species of the plants were the same.
- It may be that the plants with less water had diseases.
- It may be that the plants with 150 mL of water were also exposed to more sunlight and nutrients.
- It may be that Jasper forgot to water the plants for one day.
- It may be that Jasper used sparkling water instead of tap water.”

#### 6.2.4. Designing Experiments

Short descriptions of a scenario and hypothesis were presented, and for the multiple-choice items, participants were asked to answer questions regarding experimental designs, such as “Which of the would be the BEST dependent variable for testing this hypothesis?” and “which of the following would be the BEST experimental design for an experiment testing this hypothesis?”.

For the free-response items, participants were asked to design an experiment that could adequately test the hypothesis, as in Study 1. Here, however, is an example of a Designing Experiments item converted to multiple-choice format:

“Devon is interested in making her seeds sprout faster. She hypothesizes that higher temperatures, up to a point, can make the seeds sprout faster and earlier. However, Devon is not sure how to properly design an experiment to test this hypothesis.

Which of the would be the BEST dependent variable for testing this hypothesis?

- Rate of seed growth compared for each month between January and December of a given year.
- Rate of seed growth compared for odd versus even numbered weeks of the year.
- Rate of seed growth for extremely hot days versus extremely cold days.
- Rate of seed growth for weeks below 40 degrees Fahrenheit versus above 80 degrees Fahrenheit.
- Rate of seed growth for weeks according to their varying average temperatures.”

#### 6.2.5. Drawing Conclusions

A short description of an experiment’s results was presented, and for the multiple-choice items, participants were asked to answer questions such as “Choose the best description for this conclusion” and “Which of the following would be the BEST reason of why this conclusion is NOT correct?”.

For the free-response items, participants were asked to explain whether or not the conclusions drawn from the results were valid, as in Study 1. Here, however, is an example of a Drawing-Conclusions item in multiple-choice format:

“Tony wanted to see whether exercising for 30 min per day would reduce cholesterol levels. He had 10 subjects exercise for at least 30 minutes per day for three weeks and 10 subjects not exercise at all. He also controlled the number of calories all subjects consumed daily. He found that cholesterol levels were reduced for those who did the exercise. He concluded that exercising reduces cholesterol levels and thus lowers the risk of having heart disease.

Choose the best description for this conclusion.

F. The conclusion is correct because the connection to heart disease was proven.
G. The conclusion is incorrect because the dependent variable has no connection to the independent variable.
H. The conclusion is correct because it follows the cholesterol-levels data.
I. Tony did a great job at controlling the number of calories all subjects consumed daily.
J. The conclusion is incorrect because the number of subjects in this experiment was too small to draw meaningful conclusions.”

#### 6.2.6. Demographic Questionnaire

A demographic questionnaire assessed variables including gender, age, major, GPA, SAT and ACT scores, number of lab course taken, number of math and science courses taken, whether or not have taken research-methods courses, and whether or not contemplating career in a STEM-related field.

### 6.3. Design

Half of the students received Form A and half received Form B. All students received all item scenarios, but half the students received a given item scenario via free response and the other half received that same scenario via multiple-choice. Thus, all students received all items, but only in one or the other format, with the item format counterbalanced across the two forms. Students were randomly assigned to a form. In the analysis of variance, the independent variable was participant sex, and the dependent variables were scored on the various tests. The independent variables in the correlational analysis were Scientific-Reasoning scores, and the dependent variables were standardized ability-test scores.

### 6.4. Procedure

The participants were first asked to read and sign an informed-consent form. Then they received the Scientific-Reasoning test of either Form A or Form B. The assessments in the survey were arranged in the following order: Letter Sets, Number Series, Generating Hypotheses, Generating Experiments, Drawing Conclusions, and a brief demographic questionnaire. The first two assessments had a seven-minute time limit; they were timed by an experimenter with a stopwatch. After completion of the Number-Series test, the participants were asked to read the instructions carefully and finish the rest of the questionnaires at their own pace. After completing the survey, the participants were asked to return the survey to the experimenter, so they could receive compensation in the form of extra credit for their classes. The entire session lasted at most 1.5 h. At the end, participants received written debriefing.

### 6.5. Results

All data analyses were done via SPSS. The Letter-Sets and Number-Series items were the same as in the previous study, and reliabilities for Study 1 were provided above (under Study 1). In this study, Letter Sets had a coefficient alpha of 0.59 and Number Series a coefficient alpha of 0.65, both values very close to those in Study 1. However, the scientific-reasoning items were not the same. The Spearman-Brown corrected reliability of the Scientific-Reasoning items was 0.44, which is obviously less than satisfactory. This means that the maximum validity correlations we could obtain were at the level of 0.66 (the square root of 0.44). (Another measure of reliability, Guttman Lambda [6], corrected by the Spearman-Brown formula, came out at the same general level of reliability: 0.51.)^1^.

#### 6.5.1. Basic Statistics

Basic statistics by form are shown in Table 9.

#### 6.5.2. Comparability of Forms A and B

We first performed a series of *t-*tests to discover whether the two forms of the test, A and B, differed from each other. Recall that each item was administered either as a free-response item or as a multiple-choice item, with one form of the item appearing on Form A and the other form on Form B. Both Form A and B contained equal numbers of free-response and multiple-choice items.

The results were, for Generating Hypotheses, free response, *t* (137) = −0.99, *p =* 0.32; Generating Hypotheses, multiple choice, *t* (137) = −1.13, *p =* 0.26; for Generating Experiments, free response, *t* (137) = −0.74, *p =* 0.46; for Generating Experiments, multiple choice, *t* (137) = −0.44, *p* = 0.66; for Drawing Conclusions, free response, *t* (137) = −2.75, *p =* 0.01; for Drawing Conclusions, multiple choice, *t* (137) = 4.78, *p =* 0.00; for the Combined Scale, free response, *t* (137) = −1.73, *p =* 0.09; and for the Combined Scale, multiple choice, *t* (137) = 1.91, *p =* 0.06. Thus, although the two forms did not differ significantly for the Generating-Hypotheses and Generating-Experiments scales, the forms did differ significantly for the Drawing-Conclusions scales, even if one used a stringent cutoff point for significance (0.01). As a result, in order to combine data from Forms A and B so that data from the two forms were comparable, we converted scores on each of the two forms to *z-*scores for that form, so that each form would have a mean of 0 and a standard deviation of 1.

#### 6.5.3. Gender Differences

We also did *t-*tests for gender for each of the 8 comparisons above. Only one of the eight tests we performed was statistically significant, that between men and women for Generating Hypotheses, multiple choice, *t* (137) = −2.08, *p* = 0.04. Because the other tests across gender were not significant and because we did multiple *t-*tests and the result did not reach the 0.01 level of significance, we did not pursue this difference further.

#### 6.5.4. Intercorrelations

The complete correlation matrix is shown in Table 10. We call attention here to the correlations that are critical for our hypotheses.

As one would expect, Letter Sets and Number Series, our two measures of fluid intelligence, were significantly correlated, *r =* 0.27, *p <* 0.01. Also as expected, SAT Reading and Math were correlated, *r =* 0.36, *p <* 0.01 and also, as expected from their numerical content, Number Series and SAT Math were significantly correlated, *r =* 0.46, *p <* 0.01. These results give us some confidence that our results are in line with previous results with these and similar tests.

Our free-response and multiple-choice tests, for the combined subtests, correlated significantly with each other, *r* = 0.28, *p* < 0.01, suggesting that the two formats of the tests measured related but probably not identical constructs. These results were further elucidated by other patterns of correlations, as noted below.

The SAT Reading score was significantly correlated with scores on our overall (combined Scientific Reasoning) multiple-choice test, *r =* 0.33, *p <* 0.01, but not with scores on our overall (combined Scientific Reasoning) free-response test, *r =* 0.12, NS. SAT Math did not show significant correlations with any of our tests (which were not mathematical). Both Letter Sets and Number Series correlated significantly with the overall multiple-choice test, with correlations of *r =* 0.18, *p <* 0.05, and *r =* 0.25, *p* < 0.01, respectively. Again, however, Letter Sets and Number Series did not show significant correlations with our overall, free-response tests, with correlations of *r* = 0.08, NS, and *r =* 0.05, NS, respectively. These results, taken as a whole, suggest that using multiple-choice format did indeed increase correlations with conventional multiple-choice ability tests, as predicted.

Our main interest, of course, is whether the differences between the relevant correlations are significant. For this purpose, we did significance tests of differences between correlations for common samples of subjects (i.e., within-subjects designs). We used software (http://quantpsy.org/corrtest/corrtest2.htm) to compute these significance tests. In each case, we predicted that the correlations of the multiple-choice combined Scientific-Reasoning items with the psychometric tests would be higher than the correlations of the combined free-response Scientific-Reasoning items with the psychometric tests. Hence, we used one-tailed tests of statistical significance. For the Letter Sets test, the difference between the correlations for combined free-response and multiple-choice was not statistically significant (*z* = −0.98, *p =* 0.163). For the Number-Series test, the difference between the correlations for combined free-response and multiple-choice was statistically significant (*z =* −1.98, *p =* 0.024). For SAT Reading, difference between the correlations for combined free-response and multiple-choice was statistically significant (*z =* −1.70, *p =* 0.045). And for SAT Math, the difference between the correlations for combined free-response and multiple-choice was not statistically significant (*z* = −1.09, *p =* 0.138). Thus, Number Series and SAT Reading both showed statistically significant differences between the correlations for free-response and multiple-choice, as predicted. Letter Sets and SAT Math did not. But then, SAT Math did not correlate significantly with any of our combined Scientific-Reasoning measures. These results suggest limited but not entirely consistent support for our hypothesis that multiple-choice Scientific-Reasoning items would correlate more highly with the psychometric tests than would free-response Scientific-Reasoning items. Further research would be needed with tests of higher reliability than was shown by ours (and the short Letter-Sets and Numbers-Series tests).

#### 6.5.5. Factor Analyses

We also conducted factor analyses further to illuminate our results. We did not include SAT scores because relatively few students provided SAT scores. To have included the scores would have greatly reduced our sample size for these analyses.

Table 11 shows the results of a rotated principal-components analysis on the data for the Letter Sets and Number Series psychometric tests and for our multiple-choice and free-response items. The data yielded two factors, one primarily for the psychometric tests of fluid intelligence and the other primarily for our tests of Scientific Reasoning. However, the Scientific-Reasoning items presented in multiple-choice format loaded more highly on the fluid-intelligence factor (0.33) than did the Scientific-Reasoning items presented in free-response format (−0.10). These results suggest, as did the correlational results, that converting the Scientific-Reasoning items to multiple-choice format made them more similar to the psychometric test items in terms of what they measure.

Similar results can be seen in Table 12 for the rotated principal-axis factor solution, where again the multiple-choice Scientific-Reasoning items load higher on the factor for the psychometric tests than do the free-response Scientific-Reasoning items (with loadings of 0.31 and 0.01, respectively).

Two principal components had Eigenvalues greater than 1. Component 1 had an Eigenvalue of 1.58, accounting for 39.4% of the variance in the data. Component 2 had an Eigenvalue of 1.06, accounting for 26.5% of the variance in the data. Cumulative percent variance accounted for was 65.9%.

SAT/ACT were not included because they substantially reduced the number of cases, given that listwise deletion was used in the factor analysis.

Two rotated principal factors had Eigenvalues greater than 1. Factor 1 had an Eigenvalue of 1.58, accounting for 39.4% of the variance in the data. Factor 2 had an Eigenvalue of 1.06, accounting for 26.5% of the variance in the data. Cumulative percent variance accounted for was 65.9%.

SAT/ACT were not included because they substantially reduced the number of cases given that listwise deletion was used in the factor analysis.

### 6.6. Discussion

The results of Study 2 suggested that our hypothesis was correct that converting the Scientific-Reasoning items to multiple-choice format would bring them closer to the psychometric tests as measures of fluid intelligence. Put another way, using multiple choice format makes items more like conventional psychometric tests, but less like items that measure scientists’ actual work in research (where multiple-choice reasoning is not an option).”

## 7. General Discussion

We performed two studies to replicate, extend, and test the limits of previous findings regarding an apparent dissociation between scientific-reasoning skills in psychological science and scores on standardized tests of general cognitive abilities. The previous research showed that tests of Generating Hypotheses, Generating Experiments, and Drawing Conclusions, as well as of other psychological-scientific skills (Reviewing Articles and Reasoning about Teaching), correlated and factored with each other but generally did not correlate with (or sometimes even correlated negatively with) or factor with scores on conventional tests of cognitive abilities, such as Letter Sets, Number Series, and the SAT. In Study 1, we assessed whether these findings would apply not only to psychological science but to other “hard” sciences as well. We found that our previous results replicated and extended to a far broader domain than just psychological science—that they seem to apply to all sciences (at least to those domains of science that we investigated). In Study 2, we assessed whether these findings were in part a function of the format of presentation. Although Scientific-Reasoning problems in the laboratory are open-ended, standardized tests tend to use multiple-choice format. Did the format have an effect on our results? We predicted that using a multiple-choice format would bring our tests closer to what standardized tests of general cognitive abilities measure, although it would reduce the ecological validity of the assessment. We found, indeed, that using a multiple-choice format resulted in significant correlations with some of the standardized tests (SAT Reading and Number Series). We concluded that our results are replicable and generalizable, but also that the use of multiple-choice format brings our test scores more nearly in line with those standardized tests, at the expense of ecological validity.

Our studies, like any others, had limitations. First, our sample was drawn from a population of undergraduates in a highly selective university, mostly in the natural and social sciences, so the sample was hardly representative of a national one or even a typical college one. Second, in Study 2, more students than expected did not supply SAT scores, so we were unable to use their scores in our factor analyses because of reduced numbers of subjects that would appear in such analyses. Third, we do not at this time have predictive validity data for our measures with regard to performance in graduate school or actual scientific pursuits. Fourth, our results were not entirely consistent across all measures in all conditions.

Our hope for the future is that some graduate programs in the natural and social sciences might wish to try out our measures so that we can examine their predictive validity. We further hope that others will produce similar measures against which we can validate our measures. In our current research, we are trying to go one step further in trying to assess students’ skills in differentiating between high- and low-impact studies in the sciences.

In the end, we hope that there is a future in graduate admissions and even graduate training for measures such as ours in STEM fields. We believe that, if we hope to produce a new generation of creatively, analytically, and practically adept students, we need to do more than test for analytical skills in admissions and teach analytical skills in our training. We need fully to embrace in our teaching and assessment the full range of creative, analytical, and practical skills essential for scientific success.

## Figures and Tables

**Table 1:** Study 1 mean scores and standard deviations.

	Mean	Standard Deviation
Cumulative College GPA	3.54	0.38
SAT Reading Score	708	59.27
SAT Math Score	733	68.02
Letter Sets total score	9.86	2.33
Number Series total score	10.95	2.78
Hypotheses (New) total score	6.72	2.73
Experiments (New) total score	6.57	1.67
Conclusions (New) total scores	5.93	1.59
Hypotheses (Old) total score	4.42	1.96
Experiments (Old) total score	4.35	1.10
Conclusions (Old) total score	3.90	1.19

**Table 2:** Study 1 correlations.

		1	2	3	4	5	6	7	8	9	10	11	12	13	14	15	16	17	18	19
1	Gender	1.00	0.01	0.14	0.06	0.04	0.20	−0.05	−0.09	0.06	−0.10	0.18	−0.04	0.20	0.05	−0.04	−0.19	0.07	−0.11	−0.08
2	Age	−0.01	1.00	0.86**	−0.17	0.05	0.29 *	−0.11	−0.04	0.32 **	0.32 **	−0.20	−0.14	0.03	−0.01	0.16	0.23 *	0.03	0.14	0.06
3	Year	−0.14	0.86 **	1.00	−0.10	0.04	0.25 *	−0.17	−0.05	0.43 **	0.41 **	−0.18	−0.10	0.02	−0.06	0.22 *	0.28 **	0.00	0.16	0.07
4	GPA	0.06	−0.17	−0.10	1.00	0.21	0.13	0.31 *	0.12	−0.18	−0.32 **	−0.05	0.26 *	0.37 **	0.11	0.02	−0.02	0.24 *	−0.02	0.06
5	SAT Reading	0.04	0.05	0.04	0.21	1.00	0.33 **	0.50 *	0.18	−0.19	−0.12	0.01	0.14	−0.02	0.14	0.26 *	0.16	0.20	0.16	0.06
6	SAT Math	0.20	0.29 *	0.25 *	0.13	0.33 **	1.00	0.22	−0.06	0.13	0.09	0.03	0.25	0.40 **	−0.11	0.20	0.12	−0.05	0.04	−0.04
7	ACT	−0.05	−0.11	−0.17	0.305 *	0.50 *	0.22	1.00	0.12	−0.22	−0.23	−0.06	0.43 **	0.18	−0.05	−0.23	−0.24	−0.03	−0.27	0.02
8	STEM Career	−0.09	−0.04	−0.05	0.12	0.18	−0.06	0.12	1.00	−0.50 **	−0.45 **	0.14	−0.02	−0.13	−0.07	0.02	0.04	0.08	0.19	0.07
9	STEM Courses	0.06	0.32 **	0.43 **	−0.18	−0.19	0.13	−0.22	−0.50 **	1.00	0.63 **	−0.21 *	0.11	0.13	−0.03	0.09	−0.06	−0.09	−0.10	−0.05
10	Lab Courses	−0.10	0.32 **	0.41 **	−0.32 **	−0.12	0.09	−0.23	0.45 **	0.63 **	1.00	−0.24 *	−0.02	−0.08	−0.05	0.04	0.05	−0.13	−0.10	0.08
11	Research Courses	0.18	−0.20	−0.18	−0.05	0.01	0.03	−0.06	0.14	−0.21 *	−0.24 *	1.00	−0.13	−0.09	−0.10	−0.13	0.01	−0.03	−0.03	−0.09
12	LS	−0.04	−0.14	−0.10	0.26 *	0.14	0.25	0.43 **	−0.02	0.11	−0.02	−0.13	1.00	0.42 **	0.01	0.11	0.00	−0.01	0.05	0.16
13	NS	0.20	0.03	0.02	0.37 **	−0.02	0.40 **	0.18	−0.13	0.13	−0.08	−0.09	0.42 **	1.00	−0.05	0.14	0.04	0.00	−0.06	0.00
14	Hypotheses (New)	0.05	−0.01	−0.06	0.11	0.14	−0.11	−0.05	−0.07	−0.03	−0.05	−0.10	0.01	−0.05	1.00	0.41 **	0.33 **	0.70 **	0.35 **	0.42 **
15	Experiments (New)	−0.04	0.16	0.22 *	0.02	0.260 *	0.20	−0.23	0.02	0.09	0.04	−0.13	0.11	0.14	0.41 **	1.00	0.50 **	0.43 **	0.59 **	0.49 **
16	Conclusions (New)	−0.19	0.23 *	0.28 **	−0.02	0.16	0.12	−0.24	0.04	−0.06	0.05	0.01	0.00	0.04	0.33 **	0.50 **	1.00	0.26 *	0.48 **	0.56 **
17	Hypotheses (Old)	0.07	0.03	0.00	0.243 *	0.20	−0.05	−0.03	0.08	−0.09	−0.13	−0.03	−0.01	0.00	0.70 **	0.43 **	0.26 *	1.00	0.29 **	0.26 *
18	Experiments (Old)	−0.11	0.14	0.16	−0.02	0.16	0.04	−0.27	0.19	−0.10	−0.10	−0.03	0.05	−0.06	0.35 **	0.59 **	0.48 **	0.29 **	1.00	0.32 **
19	Conclusions (Old)	−0.08	0.06	0.07	0.06	0.06	−0.04	0.02	0.07	−0.05	0.08	−0.09	0.16	0.00	0.42 **	0.49 **	0.56 **	0.26 *	0.32 **	1.00

Notes: LS refers to Letter Sets; NS refers to Number Series, * Correlation is significant at the 0.05 level (2-tailed), ** Correlation is significant at the 0.01 level (2-tailed).

**Table 3:** Study 1. Rotated principal-components matrix: New measures plus fluid-intelligence tests ^a^.

	Component	
	1	2
Hypotheses (New)	0.73	−0.09
Experiments (New)	0.82	0.17
Conclusions (New)	0.79	0.00
Letter Sets	0.02	0.83
Number Series	0.02	0.84
Extraction Method: Principal Component Analysis.		
Rotation Method: Varimax with Kaiser Normalization.		
^a^ Rotation converged in 3 iterations.		

Notes: Two principal components had Eigenvalues greater than 1. Component 1 had an Eigenvalue of 1.86, accounting for 37.1% of the variance in the data. Component 2 had an Eigenvalue of 1.41, accounting for 28.2% of the variance in the data. Cumulative percent variance accounted for was 65.3%.

**Table 4:** Study 1. Rotated principal-factor matrix: New measures plus fluid-intelligence tests ^a^.

Factor		
	1	2
Hypotheses (New)	0.52	−0.05
Experiments (New)	0.80	0.16
Conclusions (New)	0.63	0.01
Letter Sets	0.03	0.57
Number Series	0.02	0.73
Extraction Method: Principal Axis Factoring.		
Rotation Method: Varimax with Kaiser Normalization.		
^a^ Rotation converged in 3 iterations.		

Notes: Two principal factors had Eigenvalues greater than 1. Factor 1 had an Eigenvalue of 1.86, accounting for 37.1% of the variance in the data. Factor 2 had an Eigenvalue of 1.41, accounting for 28.2% of the variance in the data. Cumulative percent variance accounted for was 65.3%.

**Table 5:** Study 1. Rotated principal-component matrix: New measures plus college-admissions tests (*g-*based) ^a^.

Component		
	1	2
Hypotheses (New)	0.68	−0.11
Experiments (New)	0.82	0.32
Conclusions (New)	0.74	−0.13
SAT Reading	−0.11	0.83
SAT Math	0.24	0.71
ACT	−0.48	0.66
Extraction Method: Principal Component Analysis.		
Rotation Method: Varimax with Kaiser Normalization.		
^a^ Rotation converged in 3 iterations.		

Notes: Two principal components had Eigenvalues greater than 1. Component 1 had an Eigenvalue of 2.06, accounting for 34.3% of the variance in the data. Component 2 had an Eigenvalue of 1.68, accounting for 28.0% of the variance in the data. Cumulative percent variance accounted for was 62%.

**Table 6:** Study 1 Principal Axis Factor Analyses: New Measures Plus College-Admissions Tests (*g-*based). Rotated Principal-Factor Matrix ^a^.

Factor		
	1	2
SAT Reading	−0.14	0.74
SAT Math	0.13	0.46
ACT	−0.46	0.58
Hypotheses (New)	0.48	−0.09
Experiments (New)	0.92	0.39
Conclusions (New)	0.55	−0.11
Extraction Method: Principal Axis Factoring.		
Rotation Method: Varimax with Kaiser Normalization.		
^a^ Rotation converged in 3 iterations.		

Notes: Two principal factors had Eigenvalues greater than 1. Factor 1 had an Eigenvalue of 2.06, accounting for 34.3% of the variance in the data. Factor 2 had an Eigenvalue of 1.68, accounting for 28.0% of the variance in the data. Cumulative percent variance accounted for was 62.3%.

**Table 7:** Study 1 Rotated Principal-Components Matrix: Complete Set of Measures ^a^.

Component			
	1	2	3
Hypotheses (New)	0.69	0.03	−0.40
Experiments (New)	0.81	0.14	0.19
Conclusions (New)	0.70	−0.35	0.14
Letter Sets	0.17	0.42	0.55
Number Series	−0.08	−0.11	0.90
SAT Reading	0.02	0.87	−0.04
SAT Math	0.15	0.39	0.54
ACT	−0.30	0.73	0.25
Hypotheses (Old)	0.76	0.22	−0.35
Experiments (Old)	0.84	−0.26	0.02
Conclusions (Old)	0.81	0.01	0.28
Extraction Method: Principal Component Analysis.			
Rotation Method: Varimax with Kaiser Normalization.			
^a^ Rotation converged in 8 iterations.			

Notes: Three principal components had Eigenvalues greater than 1. Component 1 had an Eigenvalue of 3.76, accounting for 34.2% of the variance in the data. Component 2 had an Eigenvalue of 2.25, accounting for 20.4% of the variance in the data. Component 3 had an Eigenvalue of 1.48, accounting for 13.4% of variance in the data. Cumulative percent variance accounted for was 68%.

**Table 8:** Study 1 Rotated Principal-Factor Matrix: Complete Set of Measures ^a^.

Factor			
	1	2	3
Hypotheses (New)	0.63	−0.08	−0.32
Experiments (New)	0.78	0.18	0.15
Conclusions (New)	0.66	−0.26	0.13
Letter Sets	0.13	0.38	0.38
Number Series	−0.07	−0.01	0.92
SAT Reading	−0.00	0.78	−0.08
SAT Math	0.12	0.36	0.38
ACT	−0.29	0.65	0.18
Hypotheses (Old)	0.71	0.15	−0.32
Experiments (Old)	0.84	−0.23	0.03
Conclusions (Old)	0.79	0.06	0.25
Extraction Method: Principal Axis Factoring.			
Rotation Method: Varimax with Kaiser Normalization.			
^a^ Rotation converged in 5 iterations.			

Notes: Three principal components had Eigenvalues greater than 1. Component 1 had an Eigenvalue of 3.76, accounting for 34.2% of the variance in the data. Component 2 had an Eigenvalue of 2.25, accounting for 20.4% of the variance in the data. Component 3 had an Eigenvalue of 1.48, accounting for 13.4% of variance in the data. Cumulative percent variance accounted for was 68%.

**Table 9:** Study 2 Mean Scores and Standard Deviations.

	N	Mean	St. Dev.
Total Letter Sets Score	138	9.77	2.33
Total Number Series Score	138	11.08	2.57
Total Score Hypotheses, Free Response, Form A	70	7.13	2.65
Total Score Hypotheses, Free Response, Form B	69	7.61	3.03
Total Score Hypotheses, Multiple Choice, Form A	70	2.10	0.57
Total Score Hypotheses, Multiple Choice, Form B	69	2.23	0.79
Total Score Experiments, Free Response, Form A	70	7.23	1.24
Total Score Experiments, Free Response, Form B	69	7.39	1.34
Total Score Experiments, Multiple Choice, Form A	70	1.97	0.74
Total Score Experiments, Multiple Choice, Form B	69	2.03	0.80
Total Score Conclusions, Free Response, Form A	70	5.16	0.90
Total Score Conclusions, Free Response, Form B	69	5.64	1.15
Total Score Conclusions, Multiple Choice, Form A	70	2.24	0.81
Total Score Conclusions, Multiple Choice, Form B	69	1.58	0.83
Total Score HypExpConcl, Free Response, Form A	70	19.51	3.35
Total Score HypExpConcl, Free Response, Form B	69	20.64	4.25
Total Score HypExpConcl, Multiple Choice, Form A	70	6.31	1.29
Total Score HypExpConcl, Multiple Choice, Form B	69	5.84	1.61
Age	139	19.22	0.97
GPA	94	3.58	0.41
SATReading	89	719.16	56.92
SATMath	89	743.82	57.99
ACT	80	32.90	1.80
Math and Science Courses Number	139	5.47	5.39
Lab Courses Number	139	1.50	1.99

Notes: HypExpConcl refers to total score for generating hypotheses, generating experiments, and drawing conclusions.

**Table 10:** Study 2 *Z*-Score Intercorrelations.

	TotalLetterSetsScore	TotalNumberSeriesScore	ZHyp_FR	ZHyp_MC	ZExp_FR	ZExp_MC	ZConcl_FR	ZConcl_MC	ZHypExpConcl_MC	ZHypExpConcl_FR	SATReading	SATMath	ACT
TotalLetterSetsScore	1.00	0.27 **	0.10	−0.03	0.04	0.15	−0.03	0.19 *	0.18 *	0.08	0.10	0.00	0.10
TotalNumberSeriesScore	0.27 **	1.00	−0.01	0.14	0.16	0.15	−0.05	0.17 *	0.25 **	0.05	0.14	0.46 **	0.24 *
ZHyp_FR	0.10	−0.01	1.00	0.12	0.34 **	0.12	0.16	0.10	0.18 *	0.91 **	0.12	−0.03	0.00
ZHyp_MC	−0.03	0.14	0.12	1.00	0.22 *	0.10	0.05	0.05	0.54 **	0.18 *	0.227 *	0.06	0.10
ZExp_FR	0.04	0.16	0.34 **	0.216 *	1.00	0.22 **	0.05	0.07	0.26 **	0.61 **	0.02	−0.251 *	−0.10
ZExp_MC	0.15	0.15	0.12	0.10	0.22 **	1.00	0.168 *	0.17 *	0.67 **	0.22 **	0.21 *	0.04	0.14
ZConcl_FR	−0.03	−0.05	0.16	0.05	0.05	0.17 *	1.00	0.13	0.20 *	0.40 **	0.06	−0.06	0.10
ZConcl_MC	0.19 *	0.17 *	0.10	0.05	0.07	0.17 *	0.13	1.00	0.69 **	0.13	0.21 *	−0.05	0.04
ZHypExpConcl_MC	0.18 *	0.25 **	0.18 *	0.54 **	0.26 **	0.67 **	0.20 *	0.69 **	1.00	0.28 **	0.33 **	0.01	0.15
ZHypExpConcl_FR	0.08	0.05	0.91 **	0.18 *	0.61 **	0.22 **	0.40 **	0.13	0.28 **	1.00	0.12	−0.13	−0.01
SATReading	0.10	0.14	0.12	0.23 *	0.02	0.21 *	0.06	0.21 *	0.33 **	0.12	1.00	0.36 **	0.44 **
SATMath	0.00	0.46 **	−0.03	0.06	−0.251 *	0.04	−0.06	−0.05	0.01	−0.13	0.36 **	1.00	0.69 **
ACT	0.10	0.24 *	0.00	0.10	−0.10	0.14	0.10	0.04	0.15	−0.01	0.44 **	0.69 **	1.00

Notes: FR refers to free response, MC to multiple choice. HypExpConcl refers to a combined score for Generating Hypotheses, Generating Experiments, and Drawing Conclusions, * Correlation is significant at the 0.05 level (2-tailed), ** Correlation is significant at the 0.01 level (2-tailed).

**Table 11:** Study 2 Rotated Principal-components Analysis.

Rotated Component Matrix		
		Component
	1	2
TotalLetterSetsScore	0.78	0.03
TotalNumberSeriesScore	0.78	0.11
ZHypExpConcl_MC	0.33	0.73
ZHypExpConcl_FR	−0.10	0.87
Extraction Method: Principal Component Analysis.		
Rotation Method: Varimax with Kaiser Normalization.		
Rotation converged in 3 iterations.		

Notes: MC refers to multiple choice; FR refers to free response. HypExpConcl refers to the combined score for Generating Hypotheses, Generating Experiments, and Drawing Conclusions.

**Table 12:** Study 2 Rotated Principal-Axis Factor Analysis.

Rotated Principal-Axis Factor Matrix		
	Factor	
	1	2
TotalLetterSetsScore	0.42	0.09
TotalNumberSeriesScore	0.64	0.08
ZHypExpConcl_MC	0.31	0.56
ZHypExpConcl_FR	0.01	0.55
Extraction Method: Principal Axis Factoring.		
Rotation Method: Varimax with Kaiser Normalization.		
Rotation converged in 3 iterations.		

Notes: MC refers to multiple choice; FR refers to free response. HypExpConcl refers to the combined score for Generating Hypotheses, Generating Experiments, and Drawing Conclusions.

## Footnotes

1 The design of the study made computation of reliability challenging. Although there were two forms, A and B, created for counter-balancing so that each item was presented in either free-response or multiple-choice format to each participant, different participants took each form; thus, it was not possible to compute alternate-forms reliability between Forms A and B. It also did not make sense to compute internal-consistency reliabilities, because we hypothesized that the items were not, strictly speaking, unidimensional-free-response and multiple-choice items measured related but nonidentical skills. After consultation with Professor Alan Kaufman (personal communication), an expert in psychometrics, we computed a very conservative measure of reliability by correlating the two kinds of scientific-reasoning items, free-response and multiple-choice, with each other, and then correcting the correlation by the Spearman-Brown formula because any one participant answered only half the items for each item type. As the results of the study show, however, the reliabilities were sufficient to infer meaningful patterns of results, even though use of the measures for individual interpretation would require more items of a given type. In further research, more (and perhaps better) items of each type (free-response and multiple-choice) would need to be used.
